# *Zahora*, a new monotypic genus from tribe Brassiceae (Brassicaceae) endemic to the Moroccan Sahara

**DOI:** 10.3897/phytokeys.135.46946

**Published:** 2019-12-05

**Authors:** Marcus A. Koch, Claude Lemmel

**Affiliations:** 1 Centre for Organismal Studies, Dept. Biodiversity and Plant Systematics, Heidelberg University, Heidelberg, Germany Heidelberg University Heidelberg Germany; 2 Atlas Sahara, Boudenib, Morocco Unaffiliated Boudenib Morocco

**Keywords:** Brassiceae, Brassicaceae, flora of the Sahara, Morocco, new genus, *Zahora ait-atta*

## Abstract

*Zahora ait-atta* Lemmel & M.Koch, a new species from the Moroccan Sahara, is described and documented here and constitutes a monotypic new genus. The new taxon belongs to the tribe Brassiceae (Brassicaceae), and cytogenetic and phylogenetic analyses reveal that this diploid species has a remote status of Miocene origin in the northwestern Sahara Desert. We examined the morphological differences between morphologically related genera and provide photographs of the new species. The new genus may play a key role in future *Brassica*-*Raphanus* crop research since it is placed phylogenetically at the base of a generically highly diverse clade including *Raphanus sativus*, and it shows affinities to various *Brassica* species.

## Introduction

The tribe Brassiceae is among the most complex monophyletic lineages within Brassicaceae. The tribe underwent an early genome triplication (Lysak et al. 2005) affecting subsequent diversification (Arias et al. 2014) and gave rise to approximately 50 genera comprising 250 species (Huang et al. 2019; Koch et al. 2017). The entire tribe started to evolve about 23 million years ago, and centers of origin and diversity are the entire circum-Mediterranean region (Arias et al. 2014). Phylogenic analyses have identified eight clades within the tribe Brassiceae: *Vella* L., *Zilla* Forssk., *Henophyton* Coss. & Durieu, *Crambe* L., *Cakile* Mill., *Savignya* DC., “*Nigra*”, and “*Oleracea*” (Arias and Pires 2012), but several genera remain poly- and paraphyletic such as *Brassica* L. and *Diplotaxis* DC. (Arias and Pires 2012), *Raphanus* L. (Ziffer-Berger et al. 2014), or *Sinapis* L. (Arias and Pires 2012). The tribe Brassiceae is not only characterized by an ancient triploidization, but extensive hybridization and reticulate evolution may have been involved while forming numerous polyploids. The actual amount of polyploids in the entire tribe is of about 28%; and approximately 43% of the species are monocarpic (Hohmann et al. 2015), which may coincide with arid and high-temperature environments preferred by numerous species of the tribe.

The new taxon was (re)discovered in 2015 by Claude Lemmel at isolated stands near the national road between Merzouga and Taous (Morocco) close to the border with Algeria. Since then the species has been continuously monitored by the second author and has been found at various places in that region. It is likely that in February 1951 Ph. Guinet and Ch. Sauvage might have noticed the same plant species near Tafilalet, but the plants were in bloom only and fruits were missing, therefore the botanists listed the species as *Brassica* spec. and putatively unknown (Guinet and Sauvage 1954). Originally, we thought that this plant could be of recent hybrid origin, because in this area there are many wild or cultivated cabbage-related species belonging to the genera *Brassica*, *Eremophyton* Bég., *Eruca* Mill., *Moricandia* DC., *Erucaria* Mill. and *Diplotaxis*, and hybrids are often observed between genera of Brassiceae (reviewed in Warwick et al. 2009). However, the species also occurs at sites with no other Brassicaeae nearby; and Aït-atta nomadic herders, who roam with their sheep and goats in this area, told us that they knew this plant as «Zizaou n’oudad» meaning «Barbary-sheep’s cabbage» and that it grows more or less frequently in the region in some oueds (a stream-bed that remains dry except during the rainy season) depending on local and seasonal rainfall. This field evidence encouraged us to analyze in greater detail this taxon unknown to science before.

Morphological characters of the new species do not match any known generic circumscription within Brassicaceae; although the new taxon combines characters, which are typically found in members of tribe Brassiceae. Therefore, we also analyzed chromosome number and genome size to compare results with known karyotypes, and we obtained DNA sequence information for phylogenetic placement analysis and phylogenetic reconstructions.

## Material and methods

Morphological observations and measurements of the new species were carried out based on living plant material, either from the wild or cultivated at Heidelberg Botanical Garden (HEID), as well as prepared voucher specimens. Characters were measured using a dissecting microscope. Seeds were collected in the wild from the type locality (Meknés-Tafilalet/Drâa-Tafilalet: Border region with Algeria. Near Errachidia. Oued Bou-Ibourine), and grown for subsequent analysis of chromosome number (root tips) and genome size (leaf material) following protocols provided in detail with earlier studies (Hohmann et al. 2015).

Molecular analysis following the procedure of (i) DNA extraction, (ii) PCR amplification of nuclear encoded ribosomal DNA (ITS1-ITS2 region), and (iii) direct sequencing of the PCR product as it has been described earlier in detail (Karl and Koch 2013). For DNA extraction we used leaf material from the herein presented holotype.

ITS sequence information was added to a tribal-wide alignment of Brassiceae (Huang et al. 2019) and analyzed using maximum-likelihood inferences (Stamatakis 2014) with the same settings as described earlier (Huang et al. 2019). In total 193 taxa from tribe Brassiceae plus two additional outgroups have been combined with the new ITS sequence for phylogenetic analysis. The sampling, therefore, covers approximately 77% of all known species. The entire alignment is presented with Suppl. material 2. Initial phylogenetic tests have been conducted using the phylogenetic placement tool for Brassicaceae as implemented in *BrassiBase* (https://brassibase.cos.uni-heidelberg.de/; Koch et al. 2012).

In addition, plastid DNA markers *trn*L intron and *trn*L-*trn*F intergenic spacer were amplified and sequenced (Koch et al. 2017), and results have been subsequently used for megaBLAST searches (high similarity) in GenBank to identify taxa with similar plastid (maternal) genotypes.

Temporal inferences about divergence time and age of the new species have been analyzed using BEAST (Drummond et al. 2012). Here we also used the same setting as presented earlier (Huang et al. 2019) analyzing the ITS alignment (Suppl. material 2), and details can be found with Huang et al. (2019).

## Taxonomic treatment

### Zahora ait-atta

Taxon classificationPlantaeBrassicalesBrassicaceae

Lemmel & M.Koch, gen. et sp. nov.

333BDB09-7544-5BBD-A486-FA82F4911736

urn:lsid:ipni.org:names:77203326-1

#### Type.

**Figure 1.**
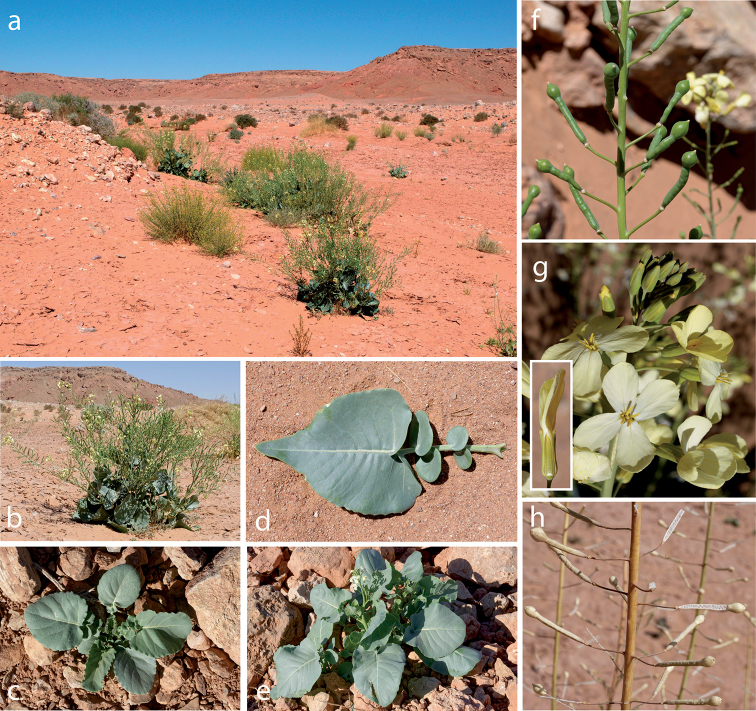
*Zahora ait-atta* in its natural environment. Border region with Algeria. Near Errachidia. Oued Bou-Ibourine – type locality **a** sandy habitat **b** flowering plant **c** rosette during winter **d** lyrate leaf from lower part of the plant **e** rosette starts building the inflorescence **f** ripening heteroarthrocarpic fruits **g** flowers and detailed view on sepals **h** siliques releasing seeds from dehiscent distal part of fruit. Images taken by C. Lemmel and Z. Attioui.

Morocco. Meknés-Tafilalet/Drâa-Tafilalet: Border region with Algeria. Near Errachidia. Oued Bou-Ibourine, « Zizaou n´oudad », gps 31.4114, -3.7220, 900 m a.s.l., 11^th^ March 2019, C. Lemmel s.n. (Holotype, HEID 505689; Isotype, G00394714, Conservatoire et jardin botanique de Genève; Paratype, HEID 505749, 505750, ex. cult. Botanical Garden Heidelberg 2019). Figure 1.

#### Description.

Herbs, woody at base, monocarpic, simple trichomes; rhizome fleshy, 2–3 cm in diam. Stems 80–140(-180) cm tall, robust, up to 1.4 cm in diam, erect, simple at base, often alternately branched in lower part. Basal leaves rosulate, fleshy; leaves lyrate, distal lobecordate, (10-)15–25(-40) cm, margin entire to distantly dentate, numerous simple trichomes on lower surface mostly along veins, upper side loosely covered with simple trichomes; cauline leaves similar but apex obtuse to weakly subacute, 10–15 × 5–7 cm. Raceme ebracteate, elongating in fruit, 40–100 cm; often branched. Sepals erect, saccate ca. 8 mm long, with few simple trichomes; petals pale-yellow,1.5–1.7 cm long, 6–7 mm wide, petal claw 8 mm long, obtuse at apex, glabrous. Filaments tetradynamous, ca. 9 mm long; nectar glands 4, rounded, elateral pair larger. Stigma entire. Infructescence with up to 100(-200) siliques, (30-)40–45(-48) mm, petiolate (9–11 mm). Fruits heteroarthrocarpic with a distal indehiscent balloon-like structure with two viable seeds (3.5–5 × 6–8 mm); proximal part dehiscent, terete (30–45 mm); 20–40 ovules; septum complete. Seeds biseriate, mucilaginous, 1.3–1.4 × 1.4–1.5 mm.

#### Etymology.

Zahora means “flower” in Arabic, indicating the attractive and peculiar appearance of the plant. “Aït-atta” are a Berber tribal confederation of south eastern Morocco who locally know the plant under the name «Zizaou n’oudad» (Barbary-sheep’s cabbage).

#### Distribution and habitat.

The species is a local endemic and was observed at the following and additional places at given dates. From these localities no additional vouchers have been collected, and to our knowledge the species has never been sampled before:

Begaa: [27^th^ January 2015] – gps 30.9453, -3.8767; 680 m a.s.l.

Khamlia: [02^nd^ February 2015] – gps 30.9895, -3.9863; 680 m a.s.l.

Oued-Bou-Ibourine: [09^th^ March 2017] – gps 31.4146, -3.7537; 900 m a.s.l.

Oued-Bou-Ibourine: [04^th^ April 2017] – gps 31.4062, -3.7353; 900 m a.s.l.

Oued-Bou-Ibourine: [02^nd^ December 2017] – gps 31.4115, -3.7214; 900 m a.s.l.

Khamlia: [08^th^ February 2018] – gps 30.9906, -3.9918; 680 m a.s.l.

Taous: [10^th^ October 2018] – gps 30.9286, -3.9753; 680 m a.s.l.

Khamlia: [08^th^ February 2019] – gps 30.99879, -3.9875; 680 m a.s.l.

Oued-Bou-Ibourine: [11^th^ March 2019] – gps 31.4114, -3.7220; 900 m a.s.l.

Oued-Bou-Ibourine: [11^th^ March 2019] – gps 31.4127, -3.7419; 890 m a.s.l.

Begaa: [12^th^ March 2019] – gps 30.9293, -3.9740; 680 m a.s.l.

**Figure 2.**
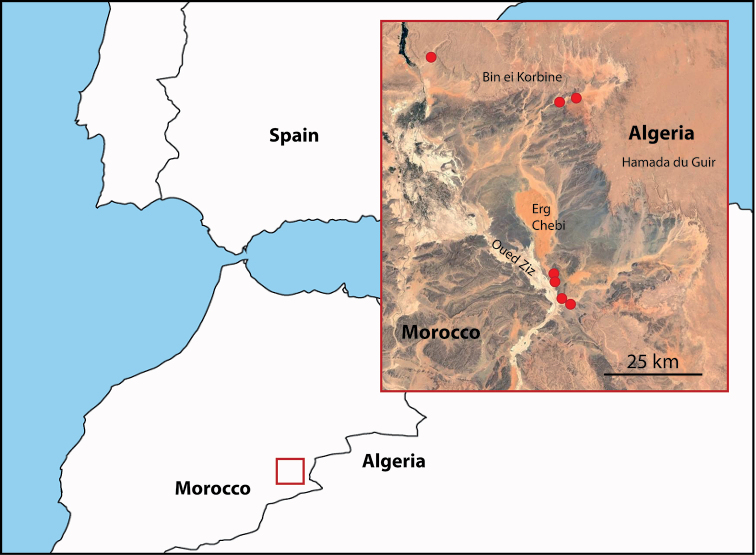
Distribution of known localities (red dots) of *Zahora ait-atta* documented from 2015 to 2019 (satellite map was taken from image metadata Copernicus/Landsat).

#### Habitat.

All places are in sandy beds of oueds flowing from the base of the kreb (cliff) of the Hamada du Guir or the Bin el Korbine.

#### Phenology.

The species flowers in spring (February to March). Seeds germinate in late summer and autumn if soil moisture is sufficient and rosettes are formed persisting throughout the winter. After fruit stage seeds are dispersed and monocarpic plants are dying.

#### Ecology.

Greenhouse and pollination experiments showed that the species is largely self-compatible. At its natural stands the plant is annual and monocarpic. However, in cultivation the plant species can be kept growing when cutting frutescence. There are two different options of seed release, either directly into a local soil seed bank from the dehiscent part of fruit or via the distal indehiscent part carrying two seeds, which may allow distributing effectively with water in the wadi systems at rare and occasional events.

#### Provisional IUCN conservation assessment.

The extent of occurrence is less than 10,000 km^2^ and falls within the limits of “Vulnerable” (VU) category under criterion B1. Since populations are of small sizes and occur at unique habitat types only, we assign an IUCN conservation status of VU B1.

## Description of characters and its discussion

In Brassicaceae there are hardly any apomorphic characters (or character states) defining genera sufficiently. This resulted in numerous poly- and paraphyletic taxa (Al-Shehbaz 2012). Therefore, we used our results from phylogenetic analysis (see below) to identify those genera «widely» associated phylogenetically with the new taxon. In total, there are 13 genera: *Brassica*, *Cordylocarpus* Desf., *Crambella* Maire, *Diplotaxis*, *Erucastrum* Gaertn., *Guiraoa* Coss., *Hirschfeldia* Moench, *Raffenaldia* Godr., *Rapistrum* Crantz, *Sinapis*, *Trachystoma* O.E.Schulz, *Morisia* J.Gray and *Otocarpus* Durieu. For entire Brassicaceae we developed a morphomatrix scoring 37 characters to deliminate genera (« morphology tool » in *BrassiBase*; https://brassibase.cos.uni-heidelberg.de/; Koch et al. 2018). This matrix builds upon an interactive key presented earlier by Ihsan Al-Shehbaz and now integrated into *BrassiBase*. We used the character matrix to identify corresponding genus-level discriminative characters (Suppl. material 1). The combination of 37 characters allow to separate the genus from 12 out of the 14 genera with the following character states found in *Zahora*: (1) annual, (2) herb, (3) glandular hairs are absent, (4) trichomes are simple, (5) stem thorns are absent, (6) basal leaves are rosette-forming, (7) leaf margin is pinnately lobed, (8) stem leaves are present, and (9) petiolate, (10) stem leaves are entire to sinuate, (11) leaf thorns are absent, (12) raceme is ebracteate, (13) petals are distinctly longer than sepals (14) petals are (pale) yellow, (15) petal are wide-shaped in upper part, (16) petal apex is obtuse, (17) petal margin is entire, (18) sepals are erect, and (19) free, (20) stamen number is 6, (21) lower part of filaments and petal claws are without any structure, and (22) filaments are free, (23) flower symmetry is actinomorphic, (24) fruit type is a silique, (25) fruits are terete, (26) fruit wall is thin and leathery, (27) fruit is dehiscent (at least in proximal part), (28) gynophore in fruit is absent, (29) septum in mature fruits is complete, (30) stigma is entire, (31) fruit appendices are missing, (32) there are more than 20 seeds per fruit, (33) seeds are arranged biseriate in middle of fruit, (34) seed wing is missing, (35) cotyledons are conduplicate, (36) seed mucilage is present, and (37) fruit orientation is spreading. The combination of 37 character states does not distinguish between *Brassica* and *Diplotaxis*, two polyphyletic genera. And the new genus shows the same general generic morphotype, too.

However, *Zahora* is different from both, *Brassica* and *Diplotaxis*, because of its peculiar fruit type. Occurrence of heteroarthrocarpic fruits with seeded beak have been described for *Brassica* (Gómez-Campo and Prakash 1999), but *Diplotaxis* shows some trend only towards this feature.

But in none of these cases functional seeds are constantly developed in the distal part. Neither *Brassica* nor *Diplotaxis* have been shown to contain species with heteroarthrocarpic fruits with disarticulation of the joint (Hall et al. 2011). In various heteroarthrocarpic species, a joint forms a novel separation layer such that the distal segment may separate and is dispersed independently of seeds from the proximal segment, a phenomenon referred to as disarticulation. *Zahora ait-atta* variant of heteroarthrocarpy can be defined as “proximal segment dehiscent with disarticulation of the indehiscent distal part”.

## Results and discussion

### Cytogenetics, phylogeny and biogeography

All molecular results refer to the voucher of the holotype, which served as source for the material.

The new species is diploid with 2*n*=18, and the haploid genome size (1C value) is 0.71 pg (+/- 8%). This corresponds to a genome size of approximately 553 MBp (Hohmann et al. 2014).

MegaBlast searches of plastid DNA sequences (*trn*L intron and *trn*LF intergenic spacer) searching for related maternal lineages as defined earlier (Arias and Pires 2012) revealed that the new genus belongs to the Oleracea-lineage: A query search with the *trn*LF intergenic spacer (GenBank submission ID2268434) (350 bp) revealed a query cover of 100% and 96.4% sequence similarity with *Brassica villosa* Raimondo & Mazzola and *B. oleracea* L., and 95.5% with *Raphanus raphanistrum* L.. A query search with the *trn*L intron (GenBank submission ID2268434) (318 bp) revealed a query cover of 98% and 99% sequence similarity with *Brassica napus* L., *B. rapa* L., *B. juncea* (L.) Crantz, *B. oleracea* and *Erucastrum gallicum* (Willd.) O.E.Schulz. There was no sequence identity match indicating any further close relation to known species and genera; and sequence identity is not high enough (>99%) to match any known sequence-based documented species or genetically defined genus.

There are no comprehensive phylogenetic hypotheses of the entire tribe Brassiceae based on the nuclear genome and discussed further taxonomically (e.g., Hall et al. 2011). However, it is known that plastid and nuclear phylogenies are largely incongruent to each other (Hall et al. 2011). Our herein presented ITS phylogeny (GenBank submission ID6359700) indicates a congruent pattern with plastid DNA data when referring to the phylogenetic position of the new taxon.

**Figure 3.**
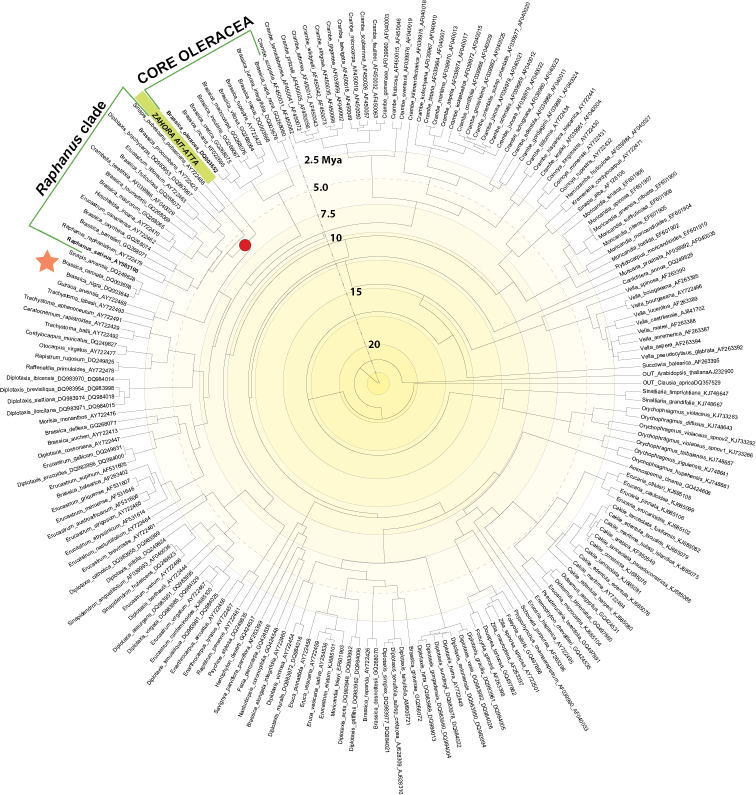
BEAST analysis of tribe Brassiceae based on ITS DNA sequence data (Suppl. material 2). The new genus *Zahora* is highlighted, and its respective stem group node is indicated (red dot). Divergence times are given as Mya (million years ago). Agronomically important species, *Brassica oleracea* and *Raphanus sativus*, are indicated and shown with their respective clades. *Brassica nigra* and *B. carinata* are also indicated with an asterisk (orange).

The new genus and species is placed at the base of a clade consisting of species from different genera, such as *Raphanus raphanistrum* L., *Sinapis pubescens* L., *Diplotaxis brachycarpa* Godr., *Erucastrum littoreum* (Pau & font Quer) Maire, *Hirschfeldia incana* (L.) Lagr.-Foss. or *Crambella teretifolia* (Batt. & Trab.) Maire herein referred to as “*Raphanus* clade” (Fig. 3). Most species of this group are confined to the southwestern Mediterranean region, and some of them are even centered in Morocco (e.g., *Crambella teretifolia*, *Diplotaxis brachycarpa*) and started to diversify approximately 5 Mya.

The sister clade of *Raphanus* and *Zahora* comprises *Brassica nigra* and *B. carinata* (indicated with Fig. 3), an interesting finding since earlier studies split the genus *Brassica* into two evolutionary lineages, the “nigra lineage” and the “rapa/oleraceae lineage” (e.g. Yang et al. 2002). However, *Zahora* is not placed closely with the Core Oleracea clade (RaXML tree, Suppl. material 3: Fig. S1; BEAST analysis, Fig. 3), which is comprising species such as *B. oleracea*, *B. villosa* or *B. rupestris* Raf.. The Core Oleracea clade within the “rapa/oleraceae lineage” started to diverge at the end of the Miocene appr. 5–6 Mya in the southwestern Mediterranean (Arias et al. 2014). Our herein presented BEAST analysis is in full support of this finding (Fig. 3).

In conclusion, both types of markers (plastid and nuclear genome) clearly indicate (i) a distinct status as a new species, (ii) no phylogenetic affinities with any known genus, and (iii) provides some biogeographical evidence of an old ancestry in the North-Western African region at Late Miocene epoch, which has been shown as a pivotal period for triggering north African aridity and creating the Sahara desert (Zhang et al. 2014). Accordingly, our herein presented BEAST analysis support a stem group age of *Zahora* of approximately 6 Mya.

Haploid genome size is comparable to other related species (*BrassiBase*; Kiefer et al. 2014), and chromosome number of *n* = 9 is a widely found and presumably ancestral situation in several *Brassica* species assemblages (Hohmann et al. 2015). A genome-level comparison of *Raphanus sativus* and *Brassica oleracea* confirmed a *n* = 9 ancestral cytotype to both of the lineages with a split time of 7–14 Mya, which coincides with our results from BEAST analysis (Fig. 3).

Since we found *Zahora* at a basal position to the entire *Raphanus* clade, the new species may play a key role in our future understanding of both genomes, *Brassica oleracea* versus *Raphanus sativus*, representing important crop plant species.

### Evolution of heteroarthrocarpic fruit in Brassiceae

Ancestral state reconstructions were unable to determine whether disarticulation precedes or follows loss of dehiscence. Regardless, variation in types among closely related taxa is the rule. For example, within the Nigra (excluding *Coincya* Porta & Rigo ex Rouy and *Muricaria prostrata* (Desf) Desv.) and Rapa/Oleracea lineages of Brassiceae most possible fruit morphologies are present (non-heteroarthrocarpic, fully dehiscent, partially dehiscent, disarticulation, and no disarticulation) (Hall et al. 2011). Therefore, *Zahora* might be an interesting study object to investigate the evolution of dehiscent and indehiscent fruit types in Brassicacae. There is some important progress to unravel the molecular mechanisms of this important trait in Brassicaceae (Carey et al. 2019), and its ecological and evolutionary relevance has been documented recently for crucifer genera *Lepidium* L. (Sperber et al. 2017) and *Aethionema* W.T. Aiton (Lenser et al. 2016, Mérai et al. 2019).

## Conclusion

*Zahora ait-atta* is described as a new species of a new monotypic genus. *Zahora* shows a peculiar fruit feature, namely heteroarthrocarpic fruits, and the species might mediate evolutionary between Core Oleracea clade (e.g. *Brassica oleracea*, *Brassica napus*) and *Raphanus sativus* and related genera. Both represent important crop plant groups with seeds playing an enormous agronomical role. The diploid new species might, therefore, serve as important germplasm reservoir to study traits and characters in a number of Brassiceae crop plants.

## Supplementary Material

XML Treatment for Zahora ait-atta

## Supplementary materials

Supplementary material 1 Brassiceae genus-level diagnostic morpho-table Data type: DNA sequence data. https://binary.pensoft.net/file/362551This dataset is made available under the Open Database License (http://opendatacommons.org/licenses/odbl/1.0/). The Open Database License (ODbL) is a license agreement intended to allow users to freely share, modify, and use this Dataset while maintaining this same freedom for others, provided that the original source and author(s) are credited.Marcus A. Koch, Claude Lemmel

Supplementary material 2 Tribal Brassiceae ITS alignment Data type: DNA sequence data. https://binary.pensoft.net/file/362552This dataset is made available under the Open Database License (http://opendatacommons.org/licenses/odbl/1.0/). The Open Database License (ODbL) is a license agreement intended to allow users to freely share, modify, and use this Dataset while maintaining this same freedom for others, provided that the original source and author(s) are credited.Marcus A. Koch, Claude Lemmel

Supplementary material 3 Figure S1. RaXML tree of Brassiceae-wide ITS data Data type: not applicable (phylogenetic reconstruction). Explanation note: Bootstrap-support from 1000 replicates is indicated. https://binary.pensoft.net/file/362553This dataset is made available under the Open Database License (http://opendatacommons.org/licenses/odbl/1.0/). The Open Database License (ODbL) is a license agreement intended to allow users to freely share, modify, and use this Dataset while maintaining this same freedom for others, provided that the original source and author(s) are credited.Marcus A. Koch, Claude Lemmel
