# Supplementary figures and images for: Zahora, a new monotypic genus from tribe Brassiceae (Brassicaceae) endemic to the Moroccan Sahara
Source: PhytoKeys. 2019 Dec 5;135:119–31. doi: 10.3897/phytokeys.135.46946 (PMC6908512; doi:10.3897/phytokeys.135.46946)

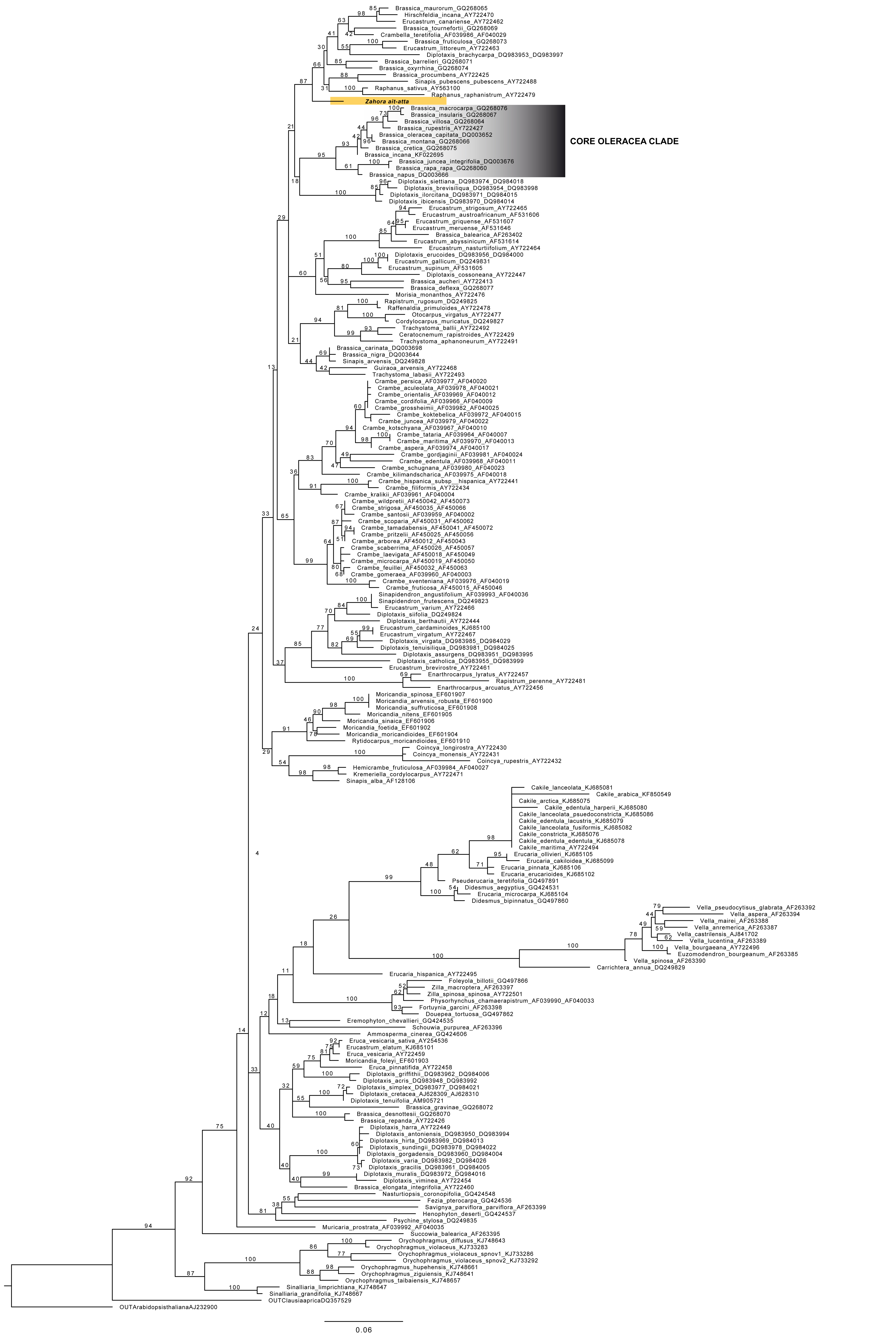

Supplement: Supplementary material 3 [file phytokeys-135-119-s003.jpg]
